# Thermal Stability Changes in Telomeric G-Quadruplex Structures Due to *N*^6^-Methyladenine Modification

**DOI:** 10.3390/epigenomes5010005

**Published:** 2021-02-02

**Authors:** Ryohei Wada, Wataru Yoshida

**Affiliations:** 1 School of Bioscience and Biotechnology, Tokyo University of Technology, 1404-1 Katakura, Hachioji, Tokyo 192-0982, Japan; b0117305d4@edu.teu.ac.jp; 2 Graduate School of Bionics, Tokyo University of Technology, 1404-1 Katakura, Hachioji, Tokyo 192-0982, Japan

**Keywords:** G-quadruplex, telomere, *N*^6^-methyladenine, thermal stability

## Abstract

*N*^6^-methyladenine modification (m^6^dA) has recently been identified in eukaryote genomic DNA. The methylation destabilizes the duplex structure when the adenine forms a Watson–Crick base pair, whereas the methylation on a terminal unpaired adenine stabilizes the duplex structure by increasing the stacking interaction. In this study, the effects of m^6^dA modification on the thermal stability of four distinct telomeric G-quadruplex (G4) structures were investigated. The m^6^dA-modified telomeric oligonucleotide d[AGGG(TTAGGG)_3_] that forms a basket-type G4 in Na^+^, d[(TTAGGG)_4_TT] that forms a hybrid-type G4 in K^+^ (Form-2), d[AAAGGG(TTAGGG)_3_AA] that forms a hybrid-type G4 in K^+^ (Form-1), and d[GGG(TTAGGG)_3_T] that forms a basket-type G4 with two G-tetrads in K^+^ (Form-3) were analyzed. Circular dichroism melting analysis demonstrated that (1) A7- and A19-methylation destabilized the basket-type G4 structure that formed in Na^+^, whereas A13-methylation stabilized the structure; (2) A15-methylation stabilized the Form-2 G4 structure; (3) A15- and A21-methylations stabilized the Form-1 G4 structure; and (4) A12-methylation stabilized the Form-3 G4 structure. These results suggest that m^6^dA modifications may affect the thermal stability of human telomeric G4 structures in regulating the biological functions.

## 1. Introduction

A human telomere consists of a tract of tandemly repeated short DNA sequences d(TTAGGG) with a single-stranded overhang at the extreme 3′ end and protective proteins [1]. As cells divide, 50 to 200 nucleotides of the telomeric region shorten, whereas the telomere length is kept in most cancer cells that over-express telomerase [2]. The single-stranded overhang can form G-quadruplex (G4) structures, which are non-canonical nucleic acid structure formed due to the stacking of G-tetrads [3]. The long telomeric oligonucleotide repeat-forms a bead-on-a-string structure in which the G4 units are connected by one TTA [4]. G4 ligands stabilize the telomeric G4 structure and inhibit the telomerase activity in cancer cells [5]; therefore, identification of factors that affect the stability of the telomeric G4 structure is important for understanding telomere biology, which can lead to applications in anti-cancer drug development.

The thermal stability of G4 structures depends on environmental factors, such as the available cations [6] and molecular crowding conditions [7]. Moreover, epigenetic DNA modifications affect the thermal stability of G4 structures. One of most analyzed epigenetic DNA modifications is 5-methylcytosine on CpG dinucleotide, which is involved in the regulation of gene expression. It has been reported that CpG methylation stabilizes *BCL-2* [8] and *VEGF* G4 structures [9,10] but destabilizes *MEST* G4 structures [11]. CpG methylation affected the binding ability of G4 structures to G4-binding proteins, which suggests that CpG methylation may regulate the biological role of G4 structures [12].

Recently, *N*^6^-methyladenine (m^6^dA) modification has been found in several eukaryotes genomic DNA, including human [13,14,15,16,17,18,19,20]. It has been reported that m^6^dA sites are enriched in exon and the higher density is associated with gene expression in human cells [19], whereas m^6^dA sites are enriched in heterochromatin regions in glioblastoma [20]. The m^6^dA modification destabilizes the duplex DNA structure, because the modification position of adenine is involved in hydrogen bond formation in Watson–Crick base pairing [21,22]. In contrast, methylation on the terminal of unpaired adenine stabilizes the duplex structure by increasing the stacking interactions [23]. These results suggest that the thermal stability of G4 structures is altered by m^6^dA modification. In particular, the *c-kit*1 G4 structure, which contains two A-G base pairs in a five-residue stem loop, was destabilized by m^6^dA modifications [24].

**Figure 1:**
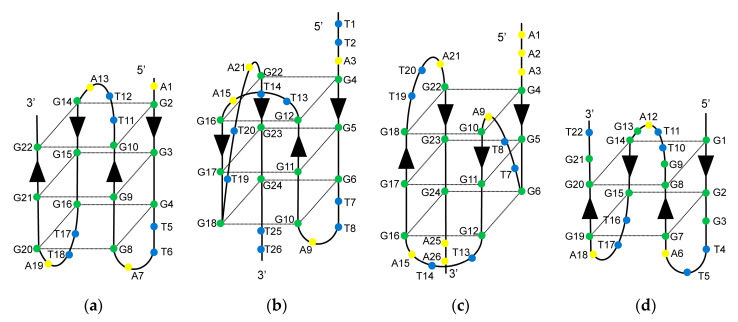
Secondary structures of the telomeric G4 structures. (**a**) Antiparallel basket-type G4 structure formed by d[AGGG(TTAGGG)_3_] (A-Tel21) in Na^+^; (**b**) Hybrid-type G4 structure (Form-2) formed by d[(TTAGGG)_4_TT] (TTA-Tel21-TT) in K^+^; (**c**) Hybrid-type G4 structure (Form-1) formed by d[AAAGGG(TTAGGG)_3_AA] (AAA-Tel21-AA) in K^+^; (**d**) Antiparallel basket-type G4 structure with two G-tetrads (Form-3) formed by d[GGG(TTAGGG)_3_T] (Tel21-T) in K^+^. Arrows indicate the DNA strand direction from 5′ to 3′.

Several distinct intramolecular G4 structures that formed by telomeric oligonucleotides have been reported. These structures contain 21-mer telomeric core sequence d[GGG(TTAGGG)_3_] in the center, and the G4 structures that formed depend on the flanking sequences and available cations [25]. In the presence of Na^+^, the wild-type of 22-mer telomeric oligonucleotide d[AGGG(TTAGGG)_3_] (A-Tel21) formed an intramolecular antiparallel basket-type G4 structure (Figure 1a) [26]. In the presence of K^+^, the wild-type of 26-mer telomeric oligonucleotide d[(TTAGGG)_4_TT] (TTA-Tel21-TT) and 25-mer telomeric oligonucleotide d[TAGGG(TTAGGG)_3_TT] formed an intramolecular hybrid-type G4 structure called Form-2/Hybrid-2 (Figure 1b) [27,28]. In contrast, the wild-type of 23-mer telomeric oligonucleotide d[TAGGG(TTAGGG)_3_] predominantly formed a distinct intramolecular hybrid-type G4 structure known as Form-1/Hybrid-1 (Figure 1c), in which the structures were determined by using mutant telomeric oligonucleotides d[TTGGG(TTAGGG)_3_A] [29], d[AAAGGG(TTAGGG)_3_AA] (AAA-Tel21-AA) [30,31], and 8-bromoguanine modified telomeric oligonucleotide [32] in the presence of K^+^. Moreover, the wild-type of 22-mer telomeric oligonucleotide d[GGG(TTAGGG)_3_T] (Tel21-T) predominantly forms an intramolecular antiparallel basket-type G4 structure with two G-tetrads, which is known as Form-3 (Figure 1d), for which the structure was determined by using 8-bromoguanine-modified telomeric oligonucleotides [33] and guanine-to-inosine substituted telomeric oligonucleotides [34].

In this study, we investigated the effect of m^6^dA modifications on the thermal stability of the telomeric G4 structures. Adenine-methylated A-Tel21, TTA-Tel21-TT, AAA-Tel21-AA, and Tel21-T were synthesized (Supplementary Materials Table S1), and the thermal stabilities were analyzed with circular dichroism (CD) spectra measurements.

## 2. Results

### 2.1. Effect of m^6^dA Modifications on the Thermal Stability of the Antiparallel Basket-Type G4 Structure That Formed in Na^+^

The d[AGGG(TTAGGG)_3_] (A-Tel21) that forms an antiparallel basket-type G4 structure in Na^+^ contained four adenines (A1, A7, A13, and A19). To analyze the effect of m^6^dA modification at each position, the oligonucleotides that contained a single-m^6^dA modification at each position were synthesized (Table S1). First, the topology was analyzed by CD spectra measurement in 100 mM Na^+^. The CD spectrum of the unmethylated A-Tel21 showed positive peaks at 244 and 297 nm and a negative peak at 263 nm, which are characteristic of the antiparallel G4 structure in 100 mM Na^+^ (Figure S1). The adenine-methylated A-Tel21 oligonucleotides also exhibited CD spectra features with an antiparallel G4 structure, which indicates that the m^6^dA modifications did not affect the topology of the G4 structure that formed in Na^+^.

**Figure 2:**
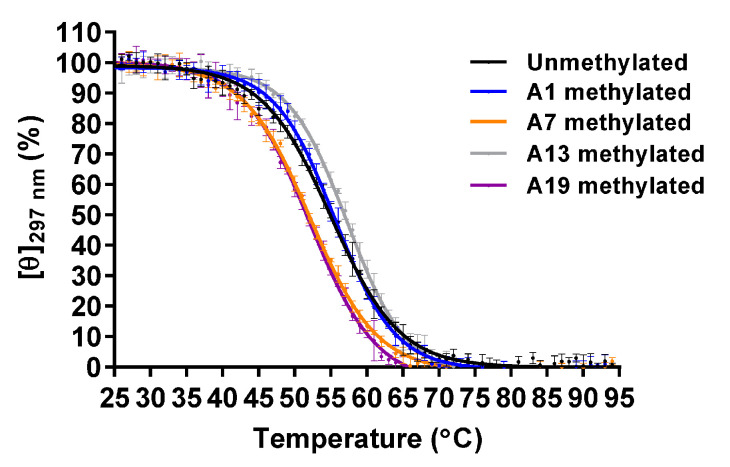
Circular dichroism (CD) melting of the unmethylated, A1-, A7-, A13- and A19-methylated G4 structures that formed by A-Tel21 (20 μM) in 7.4 mM NaH_2_PO_4_, 17.6 mM Na_2_HPO_4_, and 57.4 mM NaCl (pH 7.0) (*n* = 3; mean ± SD).

To analyze the thermal stability, CD spectra were measured from 25 to 95 °C at intervals of 1 °C, and then the melting curve was obtained using molar ellipticity at 297 nm. The CD melting curve indicated that the thermal unfolding of the G4 structure involved a two-state pathway; therefore, the melting temperature (*T*_m_) was determined to be the temperature at which normalized molar ellipticity is 50% (Figure 2). The *T*_m_ values of the unmethylated, A1-, A7-, A13-, and A19-methylated G4 structures were 54.9 ± 0.4 °C, 55.3 ± 0.4 °C, 51.9 ± 0.2 °C, 56.9 ± 0.3 °C, and 51.6 ± 0.2 °C, respectively (Table 1). Native polyacrylamide gel electrophoresis (PAGE) analysis indicated that these oligonucleotides possessed a folded monomer structure (Figure S2). These results demonstrated that A7- and A19-methylation decreased the thermal stability of the G4 structure, whereas A13-methylation increased it.

**Figure 3:**
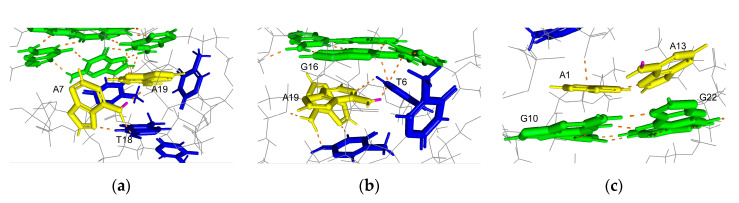
Position of adenines in the G4 structure formed by A-Tel21 in Na^+^ (PDB number: 143D). Adenine, guanine, thymine, and hydrogen bonds are shown in yellow, green, blue, and orange, respectively. Hydrogen atoms on N6, which is located on the N1 side of adenine, are shown in pink. (**a**) A7; (**b**) A19; (**c**) A13.

*N*^6^-monomethyladenine exists in two isomers, depending on the location of the methyl group on N6 in solution [21,22]. The first is a syn isomer for which the methyl group is located on the N1 side, and the other is an anti-isomer for which the methyl group is located on the N7 side of the base. The syn isomer is more stable than the anti-isomer; therefore, a hydrogen bond formation involving a hydrogen atom at N6, which is on the N1 side of the base, is inhibited by methylation. A solution structure for the antiparallel basket-type G4 structure that formed by A-Tel21 in Na^+^ has been revealed by nuclear magnetic resonance (NMR) [26]. A hydrogen atom at N6 on A7, which is located on the N1 side of the base, is involved in a hydrogen bond formation in four out of six structures (Figure 3a). Similarly, the hydrogen atom is involved in a hydrogen bond formation in five out of six structures on A19 (Figure 3b). These results indicate that m^6^dA modifications at A7 and A19 would disrupt these hydrogen bond formations, thereby reducing the thermal stability of the G4 structure. A hydrogen bond is formed on A13 in only one out of six structures, which suggests that the hydrogen atom at N6 is not significantly involved in hydrogen bond formation. In contrast, A13 stacks with G22, which is involved in a G-quartet formation (Figure 3c). The m^6^dA modification increases the stacking interaction [23]; therefore, m^6^dA modification at A13 would enhance the stacking interactions between A13 and G22 to increase the thermal stability of the G4 structure.

### 2.2. Effect of m^6^dA Modifications on the Thermal Stability of the Hybrid-Type G4 Structures Formed in K^+^

In the presence of K^+^, d[(TTAGGG)_4_TT] (TTA-Tel21-TT) forms a Form-2 G4 structure, whereas d[AAAGGG(TTAGGG)_3_AA] (AAA-Tel21-AA) forms a Form-1 G4 structure. These two oligonucleotides contain four adenines as their original base (A3, A9, A15, and A21). Therefore, A3-, A9-, A15-, and A21-methylated TTA-Tel21-TT and AAA-Tel21-AA were synthesized (Table S1). Unmethylated TTA-Tel21-TT and AAA-Tel21-AA exhibited CD spectra features of a hybrid-type G4 structure with positive peaks at around 268 and 289 nm in 100 mM K^+^ (Figures S3 and S4). Adenine-methylated TTA-Tel21-TT and AAA-Tel21-AA also exhibited CD spectra features with a hybrid-type G4 structure, which indicates that the m^6^dA modifications did not affect the topology of the hybrid-type G4 structures that formed in K^+^.

**Figure 4:**
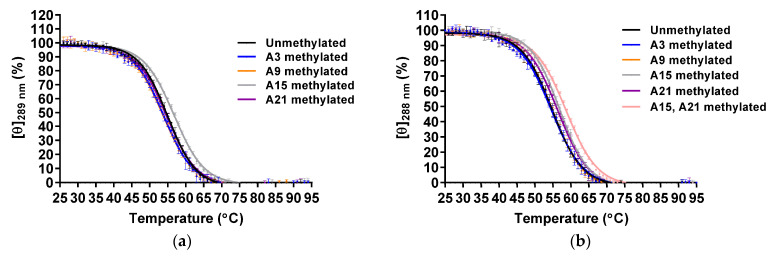
CD melting of the unmethylated and A3-, A9-, A15- and A21-methylated Form-2 (**a**) and Form-1 (**b**) G4 structures (20 μM) in 8.0 mM KH_2_PO_4_, 17.0 mM K_2_HPO_4_, and 58.0 mM KCl (pH 7.0) (*n* = 3; mean ± SD).

The thermal stabilities were analyzed by CD melting using molar ellipticities at 289 and 288 nm for the Form-2 and Form-1 G4 structure, respectively (Figure 4). The *T*_m_ values of unmethylated and A3-, A9-, A15-, and A21-methylated Form-2 G4 structures were 54.5 ± 0.3 °C, 53.5 ± 0.4 °C, 53.3 ± 0.3 °C, 56.4 ± 0.2 °C, and 54.2 ± 0.2 °C, respectively (Table 2). Native PAGE analysis indicated that these oligonucleotides folded into a monomer structure (Figure S5). These results demonstrated that A15-methylation increased the thermal stability of the Form-2 G4 structure, although other modifications did not affect them. In the case of the Form-1 G4 structure, the *T*_m_ values of the unmethylated and A3-, A9-, A15-, and A21-methylated Form-1 G4 structures were 54.0 ± 0.5 °C, 54.2 ± 0.5 °C, 54.5 ± 0.1 °C, 56.6 ± 0.3 °C, and 55.9 ± 0.3 °C, respectively (Table 2). Native PAGE analysis indicated that these oligonucleotides folded into a monomer structure (Figure S6). These results demonstrated that A15- and A21-methylations increased the thermal stability of the Form-1 G4 structure. To investigate whether A15- and A21-methylations independently stabilized the Form-1 G4 structure, both A15- and A21-methylated AAA-Tel21-AA were analyzed (Table S1). The oligonucleotide formed an intramolecular hybrid-type G4 structure (Figures S4 and S6), and the *T*_m_ value was 58.2 ± 0.2 °C (Table 2). These results demonstrated that A15- and A21-methylations independently stabilize the Form-1 G4 structure.

**Figure 5:**
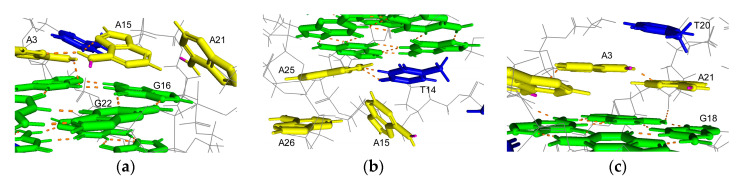
Position of adenines in the Form-2 G4 structure that formed by TTA-Tel21-TT (PDB number: 2JPZ) or in the Form-1 G4 structure that formed by AAA-Tel21-AA in K^+^ (PDB number: 2HY9). Adenine, guanine, thymine, and hydrogen bonds are shown in yellow, green, blue, and orange, respectively. Hydrogen atoms on N6, which are located on the N1 side of the adenine bond are shown in pink. (**a**) A15 in the Form-2 G4 structure; (**b**) A15 in the Form-1 G4 structure; (**c**) A21 in the Form-1 G4 structure.

In the solution structure for the Form-2 G4 structure that formed by TTA-Tel21-TT, a hydrogen atom at N6 on A15, which is located on the N7 side of the base, is involved in a hydrogen bond formation in eight out of ten structures, whereas a hydrogen atom on N6, which is located on the N1 side of the base, is involved in a hydrogen bond formation in only four out of ten structures [28]. These results suggest that m^6^dA modification at A15 would not mainly affect the hydrogen bond formation. In contrast, A15 stacks G16, which is involved in a G-quartet formation, which suggests that m^6^dA modification at A15 would enhance the stacking interactions, thereby increasing the thermal stability of the G4 structure (Figure 5a). In the solution structure of the Form-1 G4 structure that formed by AAA-Tel21-AA, hydrogen atoms at N6 on A15 and A21 are not involved in a hydrogen bond formation [31]. In contrast, A15 locates onto T14, which stacks the bottom G-quartet (Figure 5b), and A21 stacks T20 and G18, which are involved in the G-quartet formation (Figure 5c). These results suggest that m^6^dA modification at A21 would enhance stacking interactions, and m^6^dA modification at A15 may induce stacking interactions between T14 and A15.

### 2.3. Effect of m^6^dA Modifications on the Thermal Stability of the Antiparallel Basket-Type G4 Structure Formed in K^+^

In the presence of K^+^, d[GGG(TTAGGG)_3_T] (Tel21-T) predominantly forms the Form-3 G4 structure, for which the structure was determined by using modified oligonucleotides [33,34]. To investigate the effect of m^6^dA modification on Form-3, which formed by the wild-type oligonucleotide, A6-, A12-, and A18-methylated Tel21-T were analyzed (Table S1). Unmethylated Tel21-T exhibited a characteristic CD spectrum in Form-3, with two positive peaks at 251 and 292 nm in the presence of 100 mM K^+^ (Figure S7). The methylated oligonucleotides also showed characteristic CD spectra in Form-3, which indicates that m^6^dA modification did not affect the topology.

**Figure 6:**
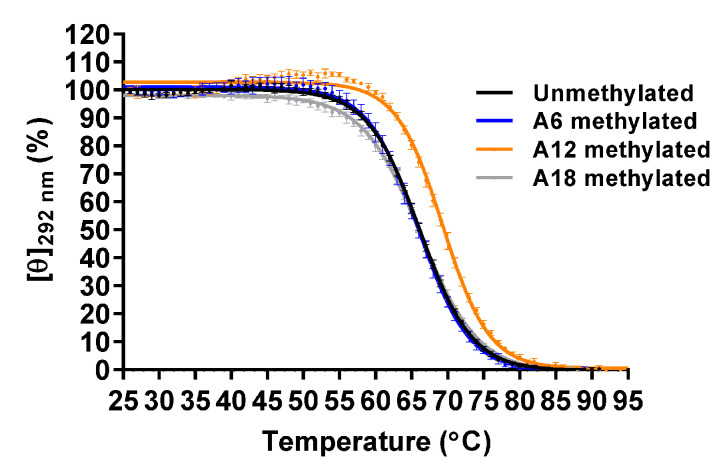
CD melting of the unmethylated and A6-, A12-, and A18-methylated Form-3 G4 structures (20 μM) in 8.0 mM KH_2_PO_4_, 17.0 mM K_2_HPO_4_, and 58.0 mM KCl (pH 7.0) (*n* = 3; mean ± SD).

The thermal stabilities were analyzed by CD melting using molar ellipticity at 292 nm. The *T*_m_ value of the unmethylated Tel21-T was 66.1 ± 0.1 °C, which was higher than that of Form-1 and Form-2, as was previously reported (Figure 6 and Table 3) [33]. The *T*_m_ values of A6-, A12-, and A18-methylated Tel21-T were 66.0 ± 0.5 °C, 69.5 ± 0.3 °C, and 66.0 ± 0.3 °C, respectively. Native PAGE analysis indicated that these oligonucleotides folded into a monomer structure (Figure S8). These results demonstrated that A12-methylation increased the thermal stability of the G4 structure, whereas A6- and A18-methylation did not affect it.

**Figure 7:**
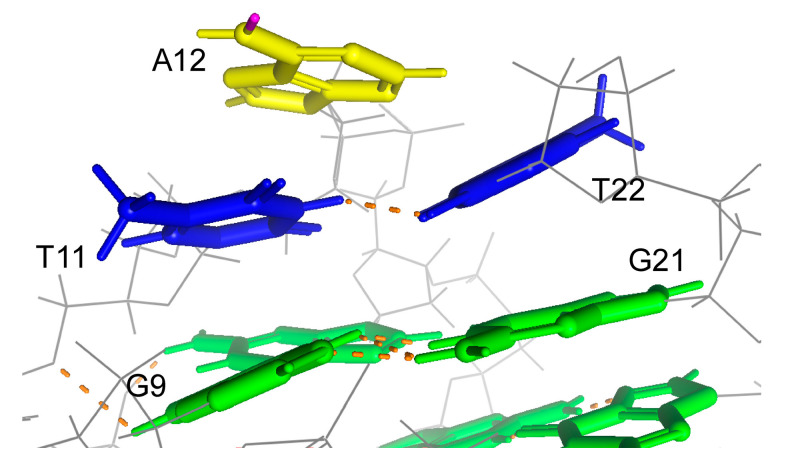
Position of A12 in the Form-3 G4 structure formed by Tel21-T, of which G7 is substituted by 8-bromoguanine in K^+^ (PDB number: 2KF7). Adenine, guanine, thymine, and hydrogen bonds are shown in yellow, green, blue, and orange, respectively. Hydrogen atoms on N6, which is located on the N1 side of the adenine bond, are shown in pink.

In the solution structure of the Form-3 G4 structure that formed by 8-bromoguanine modified Tel21-T, a hydrogen atom at N6 on A12 is involved in a hydrogen bond formation in one out of ten structures, which suggests that m^6^dA modification at A12 would not significantly affect hydrogen bond formation [33]. In contrast, A12 stacks the T11-T22 base pair, which stacks the base triple formed by G9, G13, and G21, which suggests that m^6^dA modification at A12 would enhance the stacking interactions to increase the thermal stability of the G4 structure (Figure 7).

## 3. Discussion

In this study, the effect of m^6^dA modifications on the thermal stability of four distinct telomeric G4 structures was analyzed in vitro. The A-Tel21 forms a basket-type G4 in Na^+^, whereas AAA-Tel21-AA, TTA-Tel21-TT, and Tel21-T form Form-1, Form-2, and Form-3 in K^+^, respectively. In the case of the basket-type G4 structure that formed in Na^+^, m^6^dA modifications stabilized or destabilized the G4 structure depending on the modification site. In contrast, m^6^dA modifications stabilized Form-1, Form-2, and Form-3 G4 structures. It has been reported that telomeric oligonucleotides formed Form-1, Form-2, and/or Form-3 in living human cells [35]. These results suggest that m^6^dA modifications would stabilize telomeric G4 structures in vivo.

The human telomere contains 100–200 bases of a 3′ single-stranded overhang that forms multiple G4 structures. In particular, a bead-on-a-string structure, in which the G4 units are connected by one TTA, is formed by long telomeric oligonucleotide in vitro [4]. It has been reported that long telomeric oligonucleotide containing two units of the telomeric G4-forming sequences folded into Form-1 at the 5′ end and Form-2 at the 3′ end [36], suggesting that telomeric G4 structures on long telomeric oligonucleotide would be stabilized by m^6^dA modifications. Further investigation into the effect of m^6^dA modifications on the bead-on-a-string structure would better elucidate the effect of m^6^dA modifications on the telomeric G4 structures.

The initial elongation efficiency of PCR decreased when template DNA contained G4-forming sequence of which thermal stability increased by CpG methylation [9]. There results indicated that DNA polymerase would stall on the G4 structure stabilized by CpG methylation. In this study, Form-1, Form-2, and Form-3 G4 structures were stabilized by m^6^dA modifications, suggesting that telomere elongation activity would be influenced by m^6^dA modifications.

We previously reported that the *c-kit*1 G4 structure was destabilized by m^6^dA modifications, because methylation would inhibit base-pair formation in a five-residue stem loop [24]. In contrast, Form1, Form2, and Form3 G4 structures were stabilized by m^6^dA modifications. Moreover, the thermal stability of the basket-type G4 structure formed in Na^+^ was changed depending on the position of m^6^dA modification. G4-forming sequences have been widely identified throughout the genome by means of G4 ligands with DNA microarray or high-throughput sequencing techniques [37,38,39,40]. G4-forming sequences have also been identified in mRNA [41]. The m^6^dA modification simultaneously stabilizes and destabilizes G4 structures, which suggests that m^6^dA modification may affect DNA and RNA G4 formations to regulate the biological role of G4 structures.

## 4. Materials and Methods

### 4.1. Materials

High-performance liquid chromatography–purified telomeric oligonucleotides were purchased from Tsukuba Oligo Service (Table S1). PolyT oligonucleotides were purchased from Eurofins or Integrated DNA Technologies. The other reagents were of analytical grade.

### 4.2. CD Analysis

CD spectra analyses were performed using a J1500 CD spectrometer (Jasco, Oklahoma City, OK, USA) equipped with a quartz cell with an optical path length of 1 mm. The A-Tel21 oligonucleotides were prepared at a concentration of 20 µM in 7.4 mM NaH_2_PO_4_, 17.6 mM Na_2_HPO_4_, and 57.4 mM NaCl (pH 7.0). The TTA-Tel21-TT, AAA-Tel21-AA, and Tel21-T oligonucleotides were prepared at a concentration of 20 µM in 8.0 mM KH_2_PO_4_, 17.0 mM K_2_HPO_4_, and 58.0 mM KCl (pH 7.0). The oligonucleotide solutions were heat-treated at 95 °C for 3 min and then slowly cooled down to 25 °C for 30 min by a Thermal Cycler (Thermo Fisher Scientific) before use. CD spectra were measured at a wavelength ranging from 220 to 320 nm, with a scanning speed of 200 nm/min, and a temperature ranging from 25 to 95 °C at intervals of 1 °C. For the CD melting analysis, molar ellipticities of the indicated wavelength at 25 and 95 °C were set as 100% and 0%, respectively. The normalized molar ellipticities were fitted using GraphPad Prism 7 software (GraphPad Inc.). The *T*_m_ values were recorded as the temperatures at which the normalized molar ellipticity was 50%. In all case, a *t*-test was performed and a *p*-value less than 0.005 was considered statistically significant.

### 4.3. PAGE Analysis

To conduct native PAGE analysis, the oligonucleotides were prepared as previously described. The A-Tel21 oligonucleotides (18 μL) were electrophoresed on 15% polyacrylamide gels containing 100 mM NaCl in Tris/borate buffer containing 100 mM NaCl at 40 mA for 4 h. The TTA-Tel21-TT, AAA-Tel21-AA, and Tel21-T oligonucleotides (18 μL) were electrophoresed on 15% polyacrylamide gels containing 100 mM KCl in Tris/borate buffer containing 100 mM KCl at 40 mA for 6 h. As for single-stranded DNA markers, 60 mer polyT (5.0 μg), 40 mer polyT (5.0 μg) and 20 mer polyT (1.0 μg) were electrophoresed. The oligonucleotides were visualized by ethidium bromide staining.

## Figures and Tables

**Table 1:** *T*_m_ values of the unmethylated, A1-, A7-, A13-, and A19-methylated G4 structures formed by A-Tel21 in 7.4 mM NaH_2_PO_4_, 17.6 mM Na_2_HPO_4_, and 57.4 mM NaCl (pH 7.0).

m^6^dA Site	*T*_m_ (°C)
None (unmethylated)	54.9 ± 0.4
A1	55.3 ± 0.4
A7	51.9 ± 0.2 *
A13	56.9 ± 0.3 *
A19	51.6 ± 0.2 *

*n* = 3; mean ± SD; *: *p* < 0.005 vs. unmethylated G4.

**Table 2:** *T*_m_ values of the unmethylated and A3-, A9-, A15-, and A21-methylated Form-2 and Form-1 G4 structures in 8.0 mM KH_2_PO_4_, 17.0 mM K_2_HPO_4_, and 58.0 mM KCl (pH 7.0).

m^6^dA Site	*T*_m_ (°C)	
	Form-2 G4	Form-1 G4
None (unmethylated)	54.5 ± 0.3	54.0 ± 0.5
A3	53.5 ± 0.4	54.2 ± 0.5
A9	53.3 ± 0.3	54.5 ± 0.1
A15	56.4 ± 0.2 *	56.6 ± 0.3 *
A21	54.2 ± 0.2	55.9 ± 0.3 *
A15 and A21	n.d.	58.2 ± 0.2 *

*n* = 3; mean ± SD; *: *p* < 0.005 vs. unmethylated G4; n.d.: not determined.

**Table 3:** *T*_m_ values of the unmethylated and A6-, A12-, and A18-methylated Form-3 G4 structure in 8.0 mM KH_2_PO_4_, 17.0 mM K_2_HPO_4_, and 58.0 mM KCl (pH 7.0).

m^6^dA Site	*T*_m_ (°C)
None (unmethylated)	66.1 ± 0.1
A6	66.0 ± 0.5
A12	69.5 ± 0.3 *
A18	66.0 ± 0.3

*n* = 3; mean ± SD; *: *p* < 0.005 vs. unmethylated G4.

## Data Availability

The data presented in this study are available in this article.

## Supplementary Materials

The following are available online at https://www.mdpi.com/2075-4655/5/1/5/s1, Figure S1: CD spectra of the A-Tel21 oligonucleotides in 7.4 mM NaH_2_PO_4_, 17.6 mM Na_2_HPO_4_, and 57.4 mM NaCl (pH 7.0), Figure S2: Native PAGE analysis of the A-Tel21 oligonucleotides in 100 mM Na^+^, Figure S3: CD spectra of the TTA-Tel21-TT oligonucleotides in 8.0 mM KH_2_PO_4_, 17.0 mM K_2_HPO_4_, and 58.0 mM KCl (pH 7.0), Figure S4: CD spectra of the AAA-Tel21-AA oligonucleotides in 8.0 mM KH_2_PO_4_, 17.0 mM K_2_HPO_4_, and 58.0 mM KCl (pH 7.0), Figure S5: Native PAGE analysis of the TTA-Tel21-TT oligonucleotides in 100 mM K^+^, Figure S6: Native PAGE analysis of the AAA-Tel21-AA oligonucleotides in 100 mM K^+^, Figure S7: CD spectra of the Tel21-T oligonucleotides in 8.0 mM KH_2_PO_4_, 17.0 mM K_2_HPO_4_, and 58.0 mM KCl (pH 7.0), Figure S8: Native PAGE analysis of the Tel21-T oligonucleotides in 100 mM K^+^. Table S1: Sequences of telomeric G4-forming oligonucleotides used in this study.
