# Supplementary material for: Thermal Stability Changes in Telomeric G-Quadruplex Structures Due to N6-Methyladenine Modification
Source: Epigenomes. 2021 Feb 2;5(1):5. doi: 10.3390/epigenomes5010005 (PMC8594671; doi:10.3390/epigenomes5010005)
Supplement: Supplementary file 1 [file epigenomes-05-00005-s001.pdf]

Supplementary Materials

Thermal stability changes in telomeric G-quadruplex structures due to N^6-methyladenosine modification

Ryohei Wada[1] and Wataru Yoshida[1,2,*]

1 School of Bioscience and Biotechnology, Tokyo University of Technology, 1404-1 Katakura, Hachioji, Tokyo 192-0982, Japan; b0117305d4@edu.teu.ac.jp (R.W.); yoshidawtr@stf.teu.ac.jp (W.Y.)

2 Graduate School of Bionics, Tokyo University of Technology, 1404-1 Katakura, Hachioji, Tokyo 192-0982, Japan

* Correspondence: yoshidawtr@stf.teu.ac.jp

Table S1. Sequences of telomeric G4-forming oligonucleotides used in this study

Name	Sequence (5′-3′)
A-Tel21	AGGGTTAGGGTTAGGGTTAGGG
A1-methylated A-Tel21	m^{6}dAGGGTTAGGGTTAGGGTTAGGG
A7-methylated A-Tel21	AGGGTTm^{6}dAGGGTTAGGGTTAGGG
A13-methylated A-Tel21	AGGGTTAGGGTTm^{6}dAGGGTTAGGG
A19-methylated A-Tel21	AGGGTTAGGGTTAGGGTTm^{6}dAGGG
TTA-Tel21-TT	TTAGGGTTAGGGTTAGGGTTAGGGTT
A3-methylated TTA-Tel21-TT	TTm^{6}dAGGGTTAGGGTTAGGGTTAGGGTT
A9-methylated TTA-Tel21-TT	TTAGGGTTm^{6}dAGGGTTAGGGTTAGGGTT
A15-methylated TTA-Tel21-TT	TTAGGGTTAGGGTTm^{6}dAGGGTTAGGGTT
A21-methylated TTA-Tel21-TT	TTAGGGTTAGGGTTAGGGTTm^{6}dAGGGTT
AAA-Tel21-AA	AAAGGGTTAGGGTTAGGGTTAGGGAA
A3-methylated AAA-Tel21-AA	AAm^{6}dAGGGTTAGGGTTAGGGTTAGGGAA
A9-methylated AAA-Tel21-AA	AAAGGGTTm^{6}dAGGGTTAGGGTTAGGGAA
A15-methylated AAA-Tel21-AA	AAAGGGTTAGGGTTm^{6}dAGGGTTAGGGAA
A21-methylated AAA-Tel21-AA	AAAGGGTTAGGGTTAGGGTTm^{6}dAGGGAA
A15- and A21-methylated AAA-Tel21-AA	AAAGGGTTAGGGTTm^{6}dAGGGTTm^{6}dAGGGAA
Tel21-T	GGGTTAGGGTTAGGGTTAGGGT
A6-methylated Tel21-T	GGGTTm^{6}dAGGGTTAGGGTTAGGGT
A12-methylated Tel21-T	GGGTTAGGGTTm^{6}dAGGGTTAGGGT
A18-methylated Tel21-T	GGGTTAGGGTTAGGGTTm^{6}dAGGGT

m^{6}dA: N^6-methyl-2′-deoxyadenosine

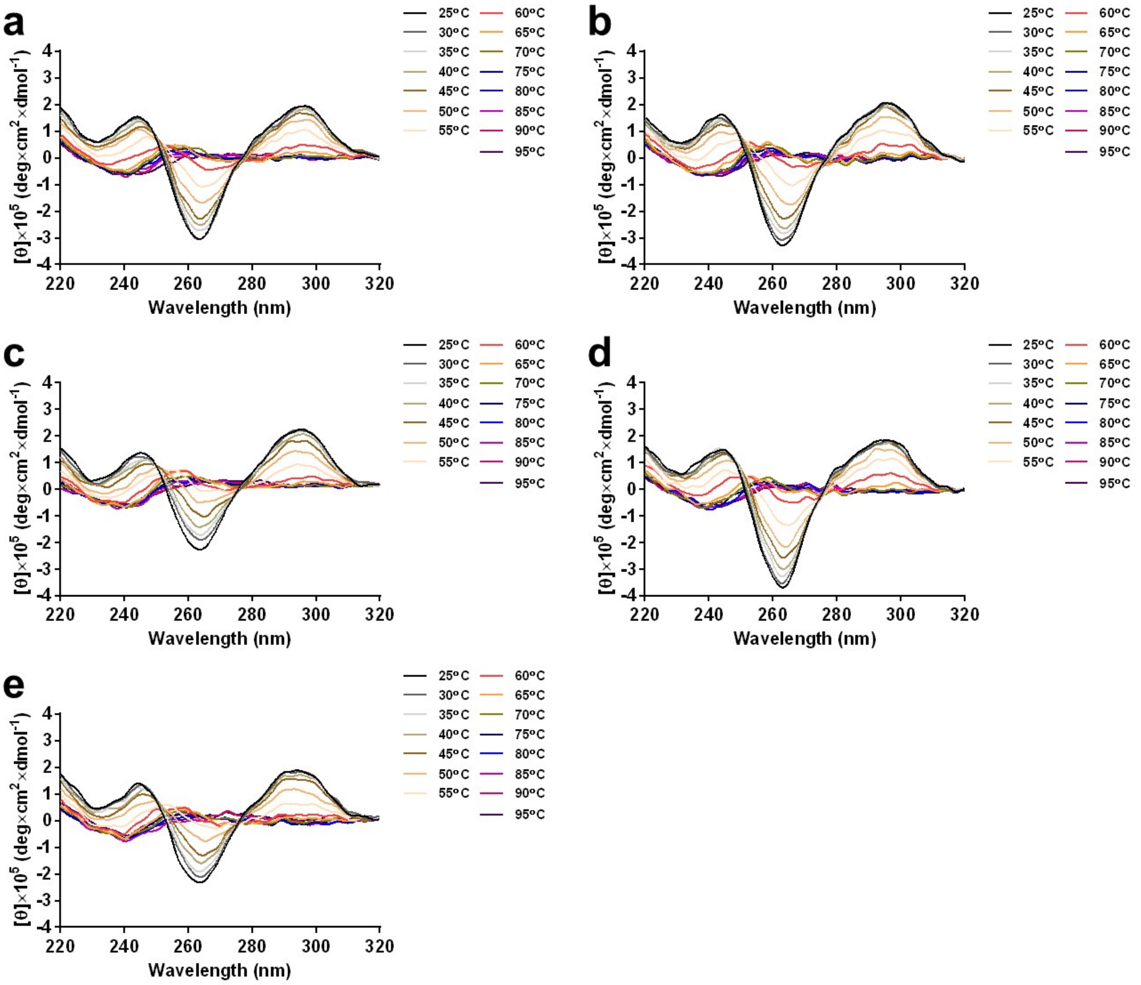

Figure S1. CD spectra of the A-Tel21 oligonucleotides in 7.4 mM NaH_2PO_4, 17.6 mM Na_2HPO_4, and 57.4 mM NaCl (pH 7.0). (a) unmethylated; (b) A1-methylated; (c) A7-methylated; (d) A13-methylated; (e) A19-methylated A-Tel21.

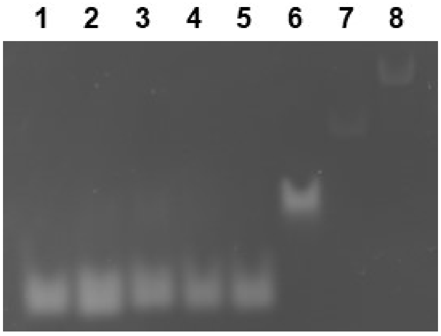

Figure S2. Native PAGE analysis of the A-Tel21 oligonucleotides in 100 mM Na^+. Lane 1, unmethylated A-Tel21; lane 2, A1-methylated A-Tel21; lane 3, A7-methylated A-Tel21; lane 4, A13-methylated A-Tel21; lane 5, A19-methylated A-Tel21; lane 6, 20-mer polyT; lane 7, 40-mer polyT; lane 8, 60-mer polyT.

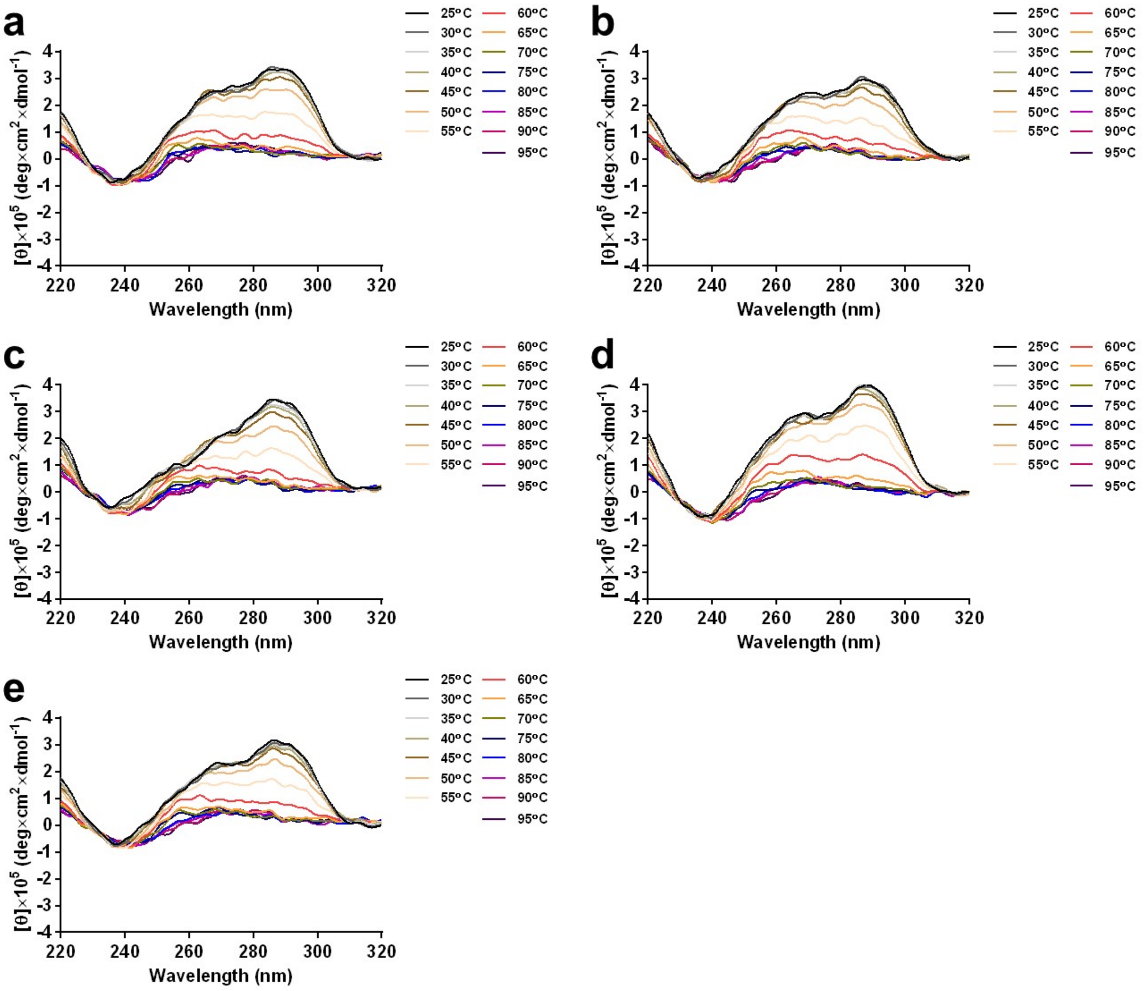

Figure S3. CD spectra of the TTA-Tel21-TT oligonucleotides in 8.0 mM KH_2PO_4, 17.0 mM K_2HPO_4, and 58.0 mM KCl (pH 7.0). (a) unmethylated; (b) A3-methylated; (c) A9-methylated; (d) A15-methylated; (e) A21-methylated TTA-Tel21-TT.

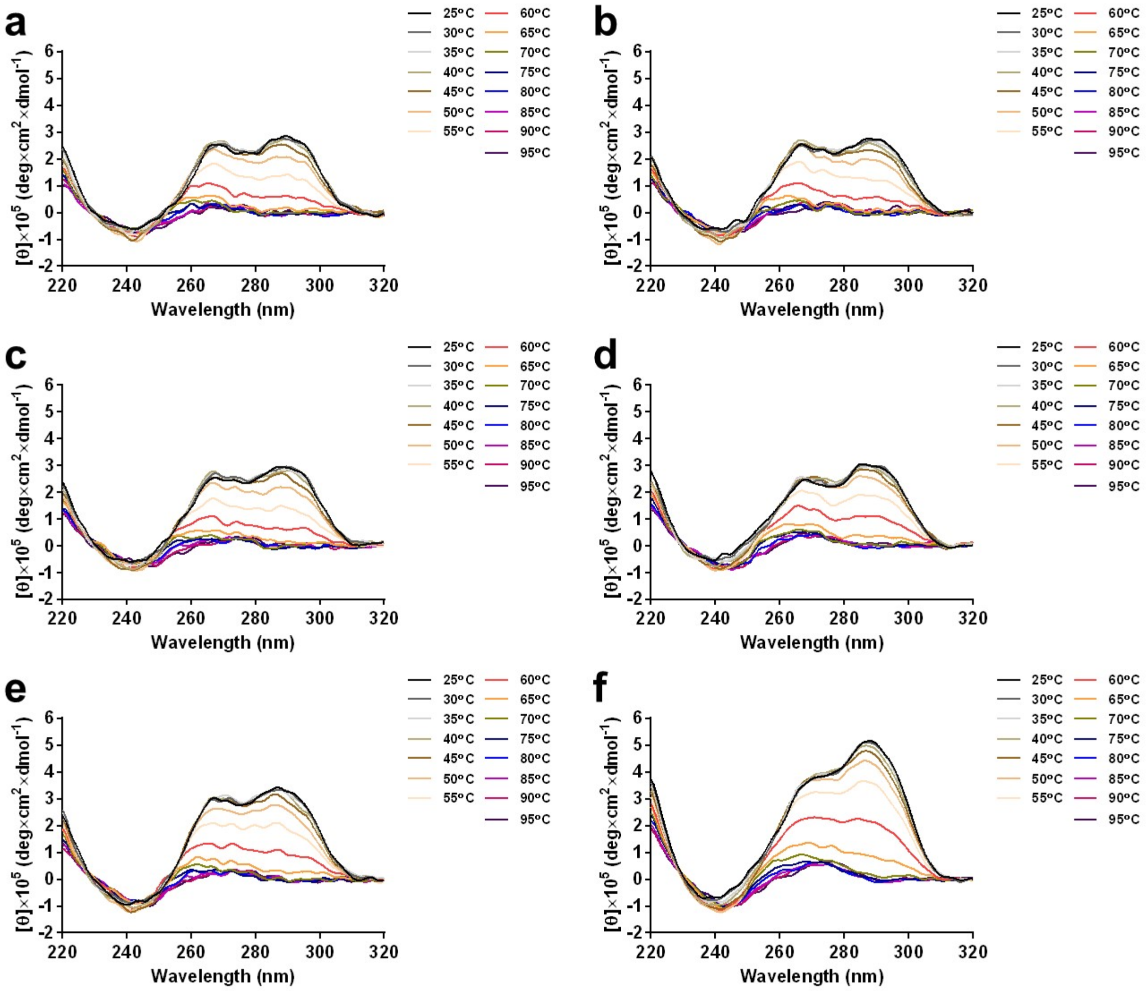

Figure S4. CD spectra of the AAA-Tel21-AA oligonucleotides in 8.0 mM KH_2PO_4, 17.0 mM K_2HPO_4, and 58.0 mM KCl (pH 7.0). (a) unmethylated; (b) A3-methylated; (c) A9-methylated; (d) A15-methylated; (e) A21-methylated AAA-Tel21-AA; (f) A15- and A21-methylated AAA-Tel21-AA.

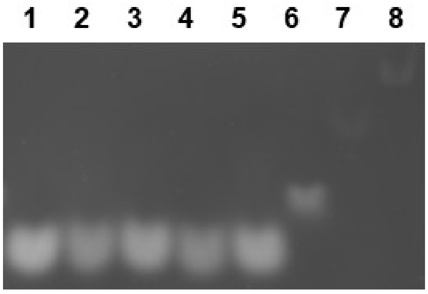

Figure S5. Native PAGE analysis of the TTA-Tel21-TT oligonucleotides in 100 mM K^+. Lane 1, unmethylated TTA-Tel21-TT; lane 2, A3-methylated TTA-Tel21-TT; lane 3, A9-methylated TTA-Tel21-TT; lane 4, A15-methylated TTA-Tel21-TT; lane 5, A21-methylated TTA-Tel21-TT; lane 6, 20-mer polyT; lane 7, 40-mer polyT; lane 8, 60-mer polyT.

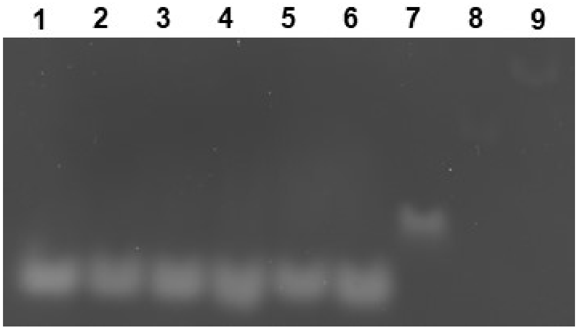

Figure S6. Native PAGE analysis of the AAA-Tel21-AA oligonucleotides in 100 mM K^+. Lane 1, unmethylated AAA-Tel21-AA; lane 2, A3-methylated AAA-Tel21-AA; lane 3, A9-methylated AAA-Tel21-AA; lane 4, A15-methylated AAA-Tel21-AA; lane 5, A21-methylated AAA-Tel21-AA; lane6, A15- and A21-methylated AAA-Tel21-AA; lane 7, 20-mer polyT; lane 8, 40-mer polyT; lane 9, 60-mer polyT.

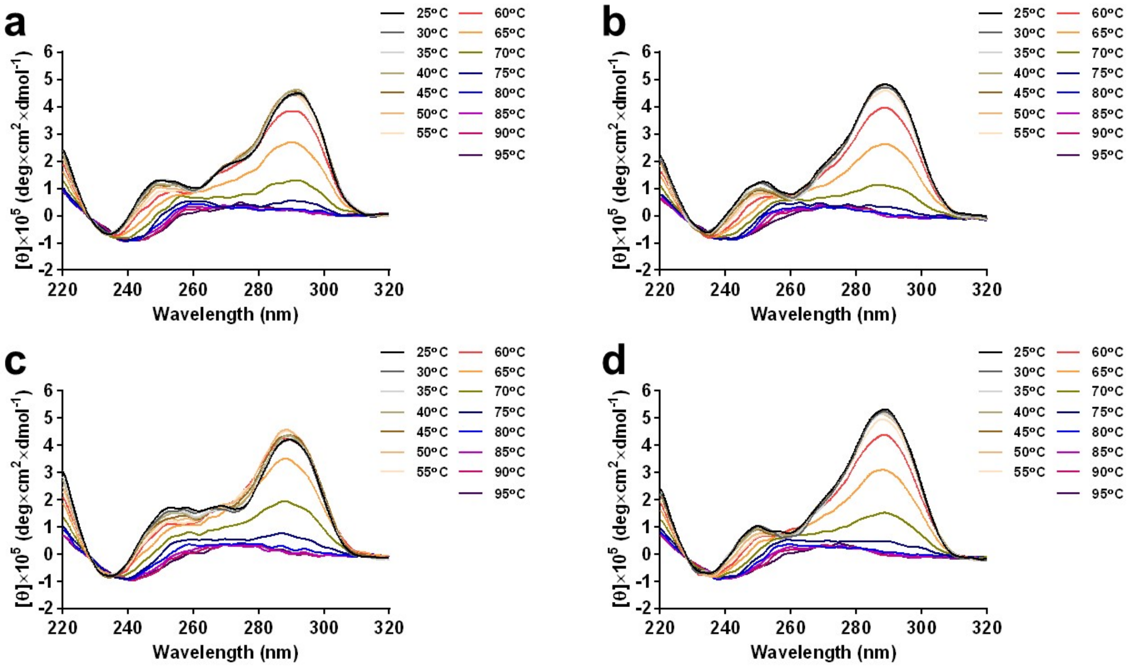

Figure S7. CD spectra of the Tel21-T oligonucleotides in 8.0 mM KH_2PO_4, 17.0 mM K_2HPO_4, and 58.0 mM KCl (pH 7.0). (a) unmethylated; (b) A6-methylated; (c) A12-methylated; (d) A18-methylated Tel21-T.

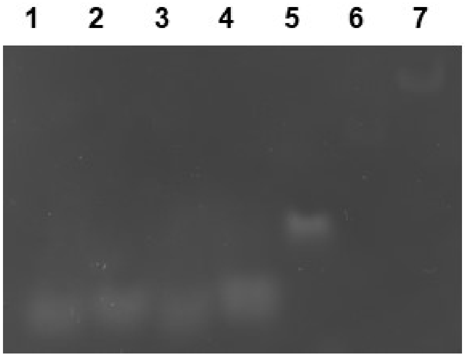

Figure S8. Native PAGE analysis of the Tel21-T oligonucleotides in 100 mM K^+. Lane 1, unmethylated Tel21-T; lane 2, A6-methylated Tel21-T; lane 3, A12-methylated Tel21-T; lane 4, A18-methylated Tel21-T; lane 5, 20-mer polyT; lane 6, 40-mer polyT; lane 7, 60-mer polyT.
